# Cardiac magnetic resonance findings in asymptomatic patients with Brugada syndrome

**DOI:** 10.1186/1532-429X-11-S1-P16

**Published:** 2009-01-28

**Authors:** Christoph J Jensen, Holger C Eberle, Dinh Q Nguyen, Thomas Schlosser, Jan Hluchy, Christoph K Naber, Georg V Sabin, Oliver Bruder

**Affiliations:** 1 Elisabeth Hospital Essen, Essen, Germany; 2 Elisabeth Hospital, Department of Cardiology and Angiology, Essen, Germany; 3 University Hospital Essen, Essen, Germany

**Keywords:** Cardiac Magnetic Resonance, Right Ventricle, Right Ventricular Outflow Tract, Brugada Syndrome, Right Ventricle Ejection Fraction

## Introduction

The Brugada syndrome (BS) is characterized by distinctive ST-segment abnormalities, malignant ventricular arrhythmias, and sudden cardiac death and is attributed to a sodium channelopathy. Additionally, right ventricular wall motion abnormalities have been described by previous studies.

## Purpose

To evaluate cardiac magnetic resonance (CMR) findings in asymptomatic patients with Brugada syndrome compared to matched controls.

## Methods

CMR was performed in 24 asymptomatic patients (13 males; mean age 42. ± 7 years) with proven Brugada syndrome on a 1.5 Tesla MR System. The imaging protocol included steady-state free precession (SSFP) cine sequences (TrueFISP, TR 3 ms, TE 1.5 ms, FA 60°, slice thickness 5 mm) in long axis views and contiguous short-axis views covering the entire left (LV) and right ventricle (RV) including the right ventricular outflow tract (RVOT). Additionally, T1 weighted turbo spin-echo sequences with and without fat suppression (TR 700 ms, TE 14 ms, FA 180°, slice thickness 5 mm) were acquired, and delayed enhancement imaging was performed following gadolinium contrast administration using segmented 2D inversion-recovery fast low angle shot sequences (TR 8 ms, TE 4 ms, FA 25°, slice thickness 5 mm) in corresponding slice orientation. Functional analysis and the area of the RVOT were calculated offline by semi-automatic post-processing software. CMR parameters were compared by Mann-Whitney test to age and sex matched controls (n = 24).

## Results

**Figure 1:**
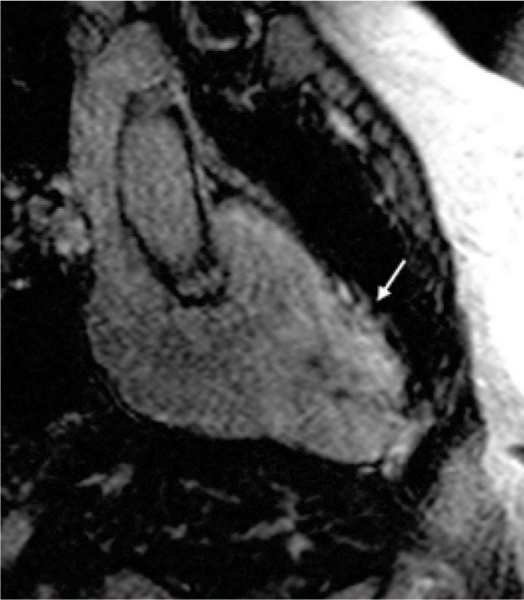
Figure 1

Patients with BS had statistically significant larger RV end-diastolic (167 ± 43 ml vs. 119 ± 24 ml, p < 0.001) and end-systolic volumes (87 ± 33 ml vs. 51 ± 16 ml, p < 0.001), lower RV ejection fraction (49 ± 8% vs. 58 ± 4%, p < 0.001) and dilated RVOT (11 ± 2 cm^2^ vs. 8 ± 1 cm^2^, p < 0.001) compared to controls. There was no statistically significant difference in LV volumes and function between patients and controls. Local RV myocardial signal enhancement in the T1 weighted turbo spin-echo images was observed in three (12.5%) patients and RV delayed enhancement in four (17%) of the 24 patients, as compared to no patient in the control group. Five (21%) patients showed localised right ventricular wall motion abnormalities, whereof in 2 patients no delayed enhancement or intramyocardial T1 signal suggesting fat deposits was present. Figure 1.

## Conclusion

Patients with proven Brugada syndrome have larger right ventricular volumes, impaired RV function and dilated RVOT compared to matched controls. Right ventricular wall motion abnormalities, localized fat deposits and delayed enhancement can be found in some of the Brugada syndrome patients indicating subtle structural heart disease.

